# Spontaneous Fusion of MSC with Breast Cancer Cells Can Generate Tumor Dormancy

**DOI:** 10.3390/ijms22115930

**Published:** 2021-05-31

**Authors:** Catharina Melzer, Juliane von der Ohe, Tianjiao Luo, Ralf Hass

**Affiliations:** Biochemistry and Tumor Biology Lab, Department of Obstetrics and Gynecology, Hannover Medical School, 30625 Hannover, Germany; catharina.melzer@gmail.com (C.M.); Ohe.Juliane.von.der@mh-hannover.de (J.v.d.O.); luo_tj@yeah.net (T.L.)

**Keywords:** mesenchymal stroma-/stem-like cells, cancer cell fusion, tumor microenvironment, cell interaction

## Abstract

Direct cellular interactions of MDA-MB-231^cherry^ breast cancer cells with GFP-transduced human mesenchymal stroma/stem-like cells (MSC^GFP^) in a co-culture model resulted in spontaneous cell fusion by the generation of MDA-MSC-hyb5^cherry GFP^ breast cancer hybrid cells. The proliferative capacity of MDA-MSC-hyb5 cells was enhanced about 1.8-fold when compared to the parental MDA-MB-231^cherry^ breast cancer cells. In contrast to a spontaneous MDA-MB-231^cherry^ induced tumor development in vivo within 18.8 days, the MDA-MSC-hyb5 cells initially remained quiescent in a dormancy-like state. At distinct time points after injection, NODscid mice started to develop MDA-MSC-hyb5 cell-induced tumors up to about a half year later. Following tumor initiation, however, tumor growth and formation of metastases in various different organs occurred rapidly within about 10.5 days. Changes in gene expression levels were evaluated by RNA-microarray analysis and revealed certain increase in dormancy-associated transcripts in MDA-MSC-hyb5. Chemotherapeutic responsiveness of MDA-MSC-hyb5 cells was partially enhanced when compared to MDA-MB-231 cells. However, some resistance, e.g., for taxol was detectable in cancer hybrid cells. Moreover, drug response partially changed during the tumor development of MDA-MSC-hyb5 cells; this suggests the presence of unstable in vivo phenotypes of MDA-hyb5 cells with increased tumor heterogeneity.

## 1. Introduction

Cell fusion in general is considered a rare event although its actual frequency may be higher as postulated by “hidden” fusions [1]. Physiological processes of cell fusion, among others, include myoblast fusion to form multinucleated myocytes in muscle fibers during muscle development, fusion of mononuclear precursor cells contributing to osteoclast formation for the maintenance, repair, and re-modelling of bone tissue; or fusion of fetal trophoblasts to syncytiotrophoblasts during the formation of placenta tissue and barrier [2,3]. Alternatively, cell fusion can occur during pathophysiological processes, such as cancer development. Indeed, cell fusion can be detected in various different cancers including leukocyte–tumor cell fusions or macrophage–tumor cell fusions, e.g., brain metastases of melanoma, lung cancer, gastric cancer, or different tumors of the breast [3,4,5,6,7,8,9]. Breast cancer cell fusion and subsequent generation of breast cancer hybrid/chimeric cells are observed during close interaction of cancer cells with adjacent cell types in the tumor microenvironment such as mesenchymal stroma/stem-like cells (MSC) [10,11,12].

Predominant locations of MSC are perivascular regions of various adult organs and tissues [13,14]. In addition, neonatal tissues such as the human umbilical cord (hUC) provide non-invasive MSC-rich sources with superior growth and expansion capacity [15]. Due to their different tissue origin and altered properties, MSC display a heterogeneous population, which is also termed multipotent stromal cells or medicinal signaling cells [16,17]. For in vitro functions, MSC exhibit adherence, migratory activity, differentiation capacity at least along mesenchymal phenotypes, and distinct core surface marker expressions such as CD73, CD90, and CD105 with simultaneous absence of at least CD14, CD31, CD34, and CD45. While these properties represent minimal characteristics of MSC as stromal cells, further specific markers or stem-like capabilities such as self-renewal capacity may only apply to a small subset within a MSC population. Accordingly, MSC heterogeneity is comprised of various subpopulations of stromal cells, stem-like cells, and cells with further special properties all displaying the minimal MSC characteristics which can be maintained during prolonged in vitro culture [18] or in a permanently proliferating human MSC-like model with reproducible properties [19]. Moreover, these minimal characteristics are further shared by more differentiated populations such as pericytes and fibroblasts. However, the properties of MSC can change in an altered environment with diverse stimuli in vitro and in vivo [20,21], which is dependent on interacting cell types. In this context, MSC predominantly interact with cancer cells within the tumor microenvironment by various indirect and direct communication mechanisms [22,23]. Strong interactions, which co-localize the two adjacent cell membranes of MSC and cancer cells in close proximity and reorganize actin-associated cytoskeletal proteins, can eventually result in the generation of corresponding hybrid/chimeric cancer cells [24,25].

Various tumor fusions including breast cancer cell fusions with MSC are described [10,26,27,28,29] whereby the resulting breast cancer hybrid cells can display altered tumorigenic activities when compared to the parental cancer cells. Within the tumor microenvironment, cancer cells can also fuse with other cell types such as endothelial cells [30]. Although precise mechanisms appear diverse among different cancer cell fusions, previous work demonstrated TNF-related signaling and apoptosis as potential triggers for breast cancer fusion with MSC [10,12,31].

MSC/breast cancer cell fusion can also occur in vivo whereby associated processes, including apoptosis and reorganization of the actin cytoskeleton, contribute to an elevated tumor plasticity and heterogeneity with a likely generation of breast cancer stem cells [24,29,32,33]. Likewise, the fusion of bone marrow derived MSC with lung cancer cells generate new cancer hybrid cell populations displaying altered properties, including cancer stem cell-like properties [34]. Moreover, hybrids from neoplastic but also from normal breast epithelial cells can display stem-like characteristics [35].

Cancer cell fusion generates chromosomal instability and aneuploidy by hybrid cell formation, whereby different polyploid cancer hybrid populations arise and increases tumor heterogeneity. Moreover, new properties of breast cancer hybrid cells also contribute to enhanced metastases [11,26,27]. The relevance of fusion processes for the generation of new cancer cell populations is underscored by the findings that MSC/breast cancer cell fusion can generate various different hybrid subtypes (e.g., MDA-MSC-hyb1 to MDA-MSC-hyb4 briefly termed MDA-hyb1 to MDA-hyb4). These cells display different tumorigenic potential, altered metastatic behavior, and changes in their sensitivity to chemotherapeutic drugs which elevates tumor heterogeneity and the potential of breast cancer stem cell expansion. In particular, human MDA-hyb1 and to a lesser extent MDA-hyb2 cancer hybrid cells represent aggressive and highly metastatic breast cancer cell lines that originated after spontaneous cell fusion of primary hUC-MSC with the MDA-MB-231 breast cancer cell line. Both MDA-hyb1 and MDA-hyb2 developed primary tumors and metastases more rapidly as compared to the parental breast cancer cells [11]. Conversely, MDA-hyb3 and MDA-hyb4 breast cancer hybrid cells were isolated following spontaneous fusion with other hUC-MSC primary cultures and these cancer hybrid populations displayed a reduced tumorigenicity when compared to the parental MDA-MB-231 cells by a markedly prolonged development of primary tumors and distant organ metastases [32].

In the present work, another breast cancer hybrid cell line is created and characterized. This demonstrates a completely different tumorigenic behavior when compared to the other current cancer hybrid populations. In contrast to an accelerated tumorigenicity of MDA-hyb1 and MDA-hyb2 and in contrast to a retarded tumorigenicity of MDA-hyb3 and MDA-hyb4, these new cancer hybrid cells exhibited a third type of tumor behavior by remaining initially quiescent and then suddenly developing tumors and metastases much faster than the parental breast cancer cells.

## 2. Results

### Introduction of MDA-MSC-hyb5 (MDA-hyb5) as a New Human Breast Cancer Hybrid Population

**Figure 1:**
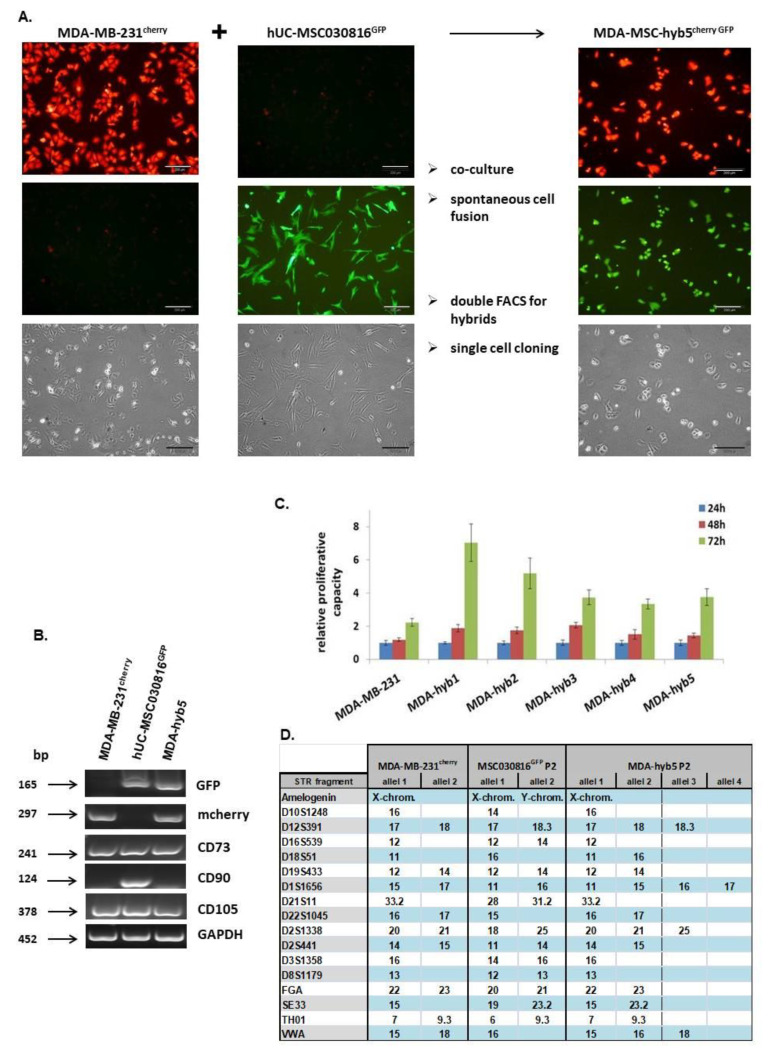
(**A**) The morphology and a preparation sequences are demonstrated for the generation and isolation of MDA-MSC-hyb5 (MDA-hyb5) cells. The upper panel represents mCherry fluorescence, the middle panel represents GFP fluorescence, and the lower panel represents the corresponding phase contrast micrographs. Bars represent 200 µm. (**B**) Gene expression in steady state cultured MDA-hyb5 cells was compared to the parental MDA-MB-231^cherry^ and hUC-MSC030816^GFP^ populations by RT-PCR, whereby GAPDH transcripts served as an equal loading control. (**C**) Measurement of proliferative capacity was performed in steady state growing cell lines of parental MDA-MB-231^cherry^ breast cancer cells and several fused hybrid populations by fluoroscan assay. Data were normalized to control cells at 24 h indicating relative fluorescence, which correlates to the relative proliferative capacity as previously described [37]. Data represents the mean ± s.d. of eight replicates, respectively. (**D**) Short tandem repeat (STR) fragment analysis of MDA-hyb5 cells was performed in comparison to the parental MDA-MB-231^cherry^ and hUC-MSC030816^GFP^ cells.

Similar to previous cancer hybrid cell isolations, a six day co-culture of 10^6^ MDA-MB-231^cherry^ breast cancer cells together with 10^6^ primary hUC-MSC030816^GFP^ P4 was performed at a ratio of 50:50, with 4500 cells/cm^2^ in MSC cell growth medium. Following the separation of this co-culture for cherry-positive, GFP-positive, and double-positive (cherry + GFP) cells by two subsequent fluorescence-activated cell sorting (FACS) (1.78 × 10^7^ total sorted cells yielded 4.24 × 10^4^ cells after 1. FACS and 3.95 × 10^3^ cells after 2. FACS), the double positive cells were subjected to single cell cloning. Over 99% of these clones died due to the aberrant fusions while a small amount of clones survived and acquired proliferative capactity during a post-hybrid selection process (PHSP). One reason for this striking effect may be the hampered cell division in most hybrid cells by uncoordinated regulatory interactions of the nuclei contents from both fusion partners which eventually resulted in cell death [36]. Single cell cloning revealed a proliferating subclone exhibiting cherry and GFP fluorescence with about 20 µm in diameter and predominantly one nucleus after 41d, which was consecutively termed MDA-MSC-hyb5 or briefly MDA-hyb5 (Figure 1A). Analysis of MDA-hyb5 cells compared to the two parental MDA-MB-231^cherry^ and hUC-MSC030816^GFP^ populations by RT-PCR revealed the appropriate expression of the fluorescent genes in the three cell types. Moreover, the MSC typical mRNA transcripts of CD73 and CD105 were detectable in all three cell lines while CD90 expression was limited to hUC-MSC and not detectable in MDA-MB-231 or MSC-hyb5 cells (Figure 1B). Continuous growth properties of MDA-hyb5 cells were compared to the parental MDA-MB-231 breast cancer cell line and to other related cancer hybrid populations such as MDA-hyb1, MDA-hyb2, MDA-hyb3, and MDA-hyb4 and they all displayed different biological properties [11,32]. MDA-hyb5 cells demonstrated a similar proliferative capacity such as MDA-hyb3 and MDA-hyb4 cells, which reached about half of the fastest growing MDA-hyb1 cells and were about 30% less than MDA-hyb2 cells. However, MDA-hyb5 growth potential was accelerate by about 1.8-fold when compared to the parental MDA-MB-231^cherry^ breast cancer cells (Figure 1C). These findings indicated MDA-hyb5 as a new human breast cancer hybrid population with more properties similar to MDA-MB-231 and an enhanced rate of cell division. Further characterization of MDA-hyb5 cells was performed by short tandem repeat (STR) fragment analysis. In comparison to the STR patterns of the parental MDA-MB-231^cherry^ and hUC-MSC030816^GFP^, the hyperploidy of MDA-hyb5 cells was detected along with the presentation of 3 and 4 alleles of distinct DNA parts (Figure 1D).

**Figure 2:**
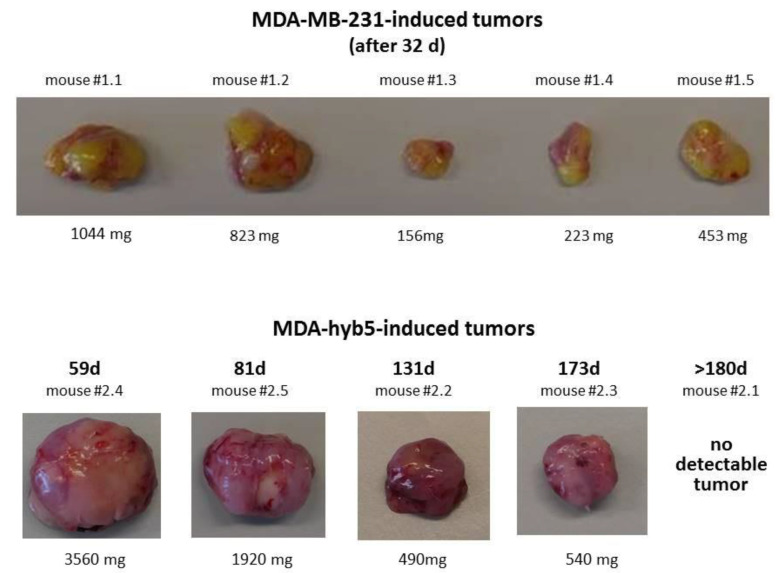
Tumors developed by subcutaneous injection of 2 × 10^6^ MDA-MB-231^GFP^ or MDA-hyb5 cells were dissected from NODscid mice at the indicated time points and weighted.

According to the different tumor-developing effects reported for other MDA-hybrid populations [11,32] we were interested in the tumorigenic behavior of MDA-hyb5 cells. Using an in vivo tumor model, subcutaneous injection was performed as a control with human MDA-MB-231^GFP^ breast cancer cells into the left shoulder of five NODscid mice where tumor development was observed in all mice and dissected after 32 days (Figure 2). A similar subcutaneous injection of MDA-hyb5 cells also developed subcutaneous tumors although at different kinetics. One out of the five mice demonstrated no detectable tumor growth within 180 days post injection (Figure 2). During the whole experiment all mice displayed a constant body weight without significant changes.

Appearance of tumors by MDA-MB-231 cells in all injected mice was consistent with observations in previous work [11]. Tumor growth became detectable between day 10 and day 18 in MDA-MB-231-induced tumors and termination was performed by cervical dislocation of the mice according to the animal welfare guidelines after 32 days. In contrast, tumor detection and development of MDA-hyb5 cells appeared at subsequent later time points (Table 1).

**Figure 3:**
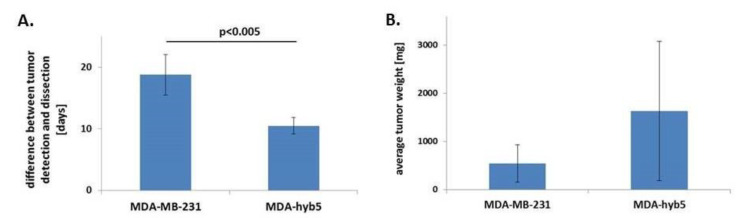
(**A**) The average time between the earliest detection of tumors and following dissection after growth of the tumors was calculated and compared between parental MDA-MB-231 cells (*n* = 5) and fusion hybrid MDA-hyb5 cells (*n* = 4). Significance (*p*) was calculated by unpaired student’s *t*-test. (**B**) The average tumor weight of MDA-MB-231- and MDA-hyb5-induced tumors was calculated by the mean ± s.d., respectively. Differences were not significant.

These data demonstrated that after initial detection, subsequent tumor growth by MDA-hyb5 cancer hybrid cells occurred within 10.5 ± 1.3 days, which is much faster by about half of the time when compared to the development of the parental MDA-MB-231-induced tumors after 18.8 ± 3.3 days (Figure 3A). Moreover, the average tumor weight was apparently higher in MDA-hyb5 tumors with 1628 ± 1448 mg as compared to 540 ± 384 mg in MDA-MB-231 tumors. However, this difference was not statistically significant (Figure 3B).

Accordingly, the average time between tumor incidence and dissection revealed a 1.8-fold faster tumor growth induced by MDA-hyb5 cells when compared to the parental MDA-MB-231 cells, whereby relative tumor weights remained within a similar range.

**Figure 4:**
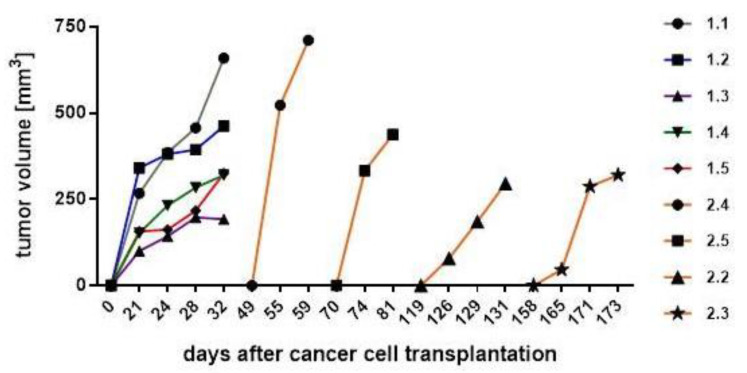
After tumor cell injection of MDA-MB-231 (mouse #1.1 to #1.5) and MDA-hyb5 (mouse #2.2 to #2.5) into the NODscid mice, the tumors first became detectable at the indicated time points. Continuous tumor growth and detection of appropriate sizes were measured using a digital caliper. Progressive tumor volumes relative to the corresponding time points were calculated with the longitudinal diameter (length) and the transverse diameter (width) in the modified ellipsoidal formula: volume = π/6 × width × (length)^2^, as previously reported [38,39].

Tumor volumes were calculated at different time points during tumor development. Thus, digital caliper measurements of MDA-MB-231 tumors revealed volumes of 192 to 659 mm^3^. Likewise, MDA-hyb5 tumors exhibited volumes between 296 to 711 mm^3^ at the final point of tumor dissection (Figure 4). As a remarkable difference, however, the original incidence of MDA-hyb5-induced tumors occurred at diverse starting time points, which were markedly delayed at least up to approximately a half year when compared to the initial MDA-MB-231-induced tumor detection. Following animal examination that was conducted twice a week after MDA-hyb5 tumor cell application, initial tumor development became detectable after 49 d, 70 d, 122 d, and 161 d, respectively and subsequent tumor growth continued for a short time until termination and tumor dissection (Figure 4).

Cumulatively, these findings indicated that the MDA-hyb5 cells remained quiescent within the mouse tissue in a dormant-like state. Once activated for tumor growth, however, MDA-hyb5 cells developed similar sized tumors 1.8-fold faster as compared to MDA-MB-231 cells.

**Figure 5:**
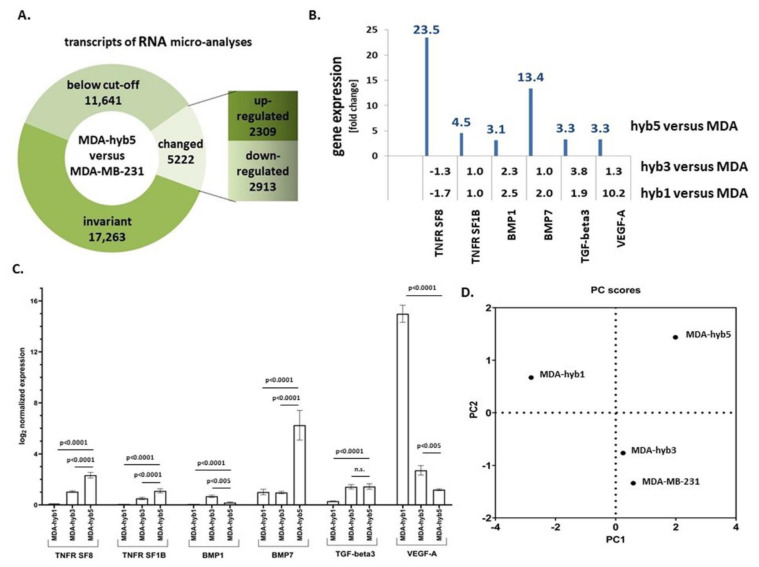
(**A**) RNA microarray analysis of 34,126 transcripts was performed in MDA-hyb5 cells as compared to the parental MDA-MB-231 cells. Changes in transcript levels below 2-fold were considered below cut-off. (**B**) Analysis of the RNA microarray data was performed with a focus on the expression of dormancy-associated genes (tumor necrosis factor receptor superfamily member 8 (TNFR SF8); tumor necrosis factor receptor superfamily member 1B (TNFR SF1B); bone morphogenic protein1 (BMP1); bone morphogenic protein7 (BMP7); transforming growth factor-beta3 (TGF-beta3); vascular endothelial growth factor-A (VEGF-A)). Differences in fold changes are indicated for the relationship of gene expression in MDA-MSC-hyb5 (hyb5), MDA-MSC-hyb3 (hyb3), and MDA-MSC-hyb1 (hyb1) cells versus the parental MDA-MB-231 (MDA) cells, respectively. (**C**) Gene expression levels were normalized to GAPDH and RPL13A by qPCR. Data represents the mean + SEM of the corrected expression levels (*n* = 4) and significance (*p*) was calculated by the Dunnett’s multiple comparison ANOVA test (ns = not significant). (**D**) A scattered plot of principal components (PC) was generated by GraphPad Prism v9.00 and demonstrated the differences between the cell lines according to the genes analyzed.

Although molecular stimuli and signals for the activation of quiescent MDA-hyb5 cells to initiate tumor growth still remain to be elucidated, RNA microarray analysis was performed for comparison with the parental MDA-MB-231 cells and for the identification of potential expression of dormancy-associated genes. From the 34,126 analyzed transcripts, 2309 mRNAs were up-regulated and 2913 mRNAs were down-regulated in MDA-hyb5 cells when compared to the parental MDA-MB-231 cells (Figure 5A). Further analysis was performed by considering the different tumor behavior of MDA-hyb1 and MDA-hyb3 cells, which developed tumors directly after the subcutaneous cancer cell injection in contrast to the initial dormancy phase of MDA-hyb5 cells. In particular, TNF (tumor necrosis factor) ligand-associated transcripts and TNF receptor-associated transcripts were markedly enhanced expressed in MDA-hyb5 as compared to MDA-MB-231 cells, in contrast to minor differences in the MDA-hyb1 versus MDA-MB-231 or the MDA-hyb3 versus MDA-MB-231 expression levels (Figure 5B). Similar results were obtained for structural genes including entactin (202-fold enhanced in MDA-hyb5), which connects the networks formed by collagens and laminins and transcripts of fibromodulin (228-fold enhanced in MDA-hyb5) participating in the assembly of collagen fibers and restructuring the actin cytoskeleton. Interestingly, previous work has demonstrated that laminin-111 in a three-dimensional cell culture system induced quiescence of breast epithelial cells by depletion of nuclear-associated actin [40,41].

Among others, gremlin-1 transcripts were 154-fold enhanced expressed in MDA-hyb5 versus MDA-MB-231, which is paralleled by little if any expression difference of MDA-hyb1 versus MDA-MB-231 or MDA-hyb3 versus MDA-MB-231 cells (NCBI-GEO database accession no. #GSE157199). Gremlin-1 transcripts are found in various different tumor types and can functionally interfere with the TGF-β (transforming growth factor-beta) signaling pathway by the inhibition of the bone morphogenetic proteins BMP2 and BMP4. These findings underscored substantial differences in the tumor behavior of instant tumor-developing MDA-hyb1 and MDA-hyb3 cells in contrast to a transient initial dormancy phase of MDA-hyb5 cells.

Different genes associated with metastasis and dormancy, such as VEGF-A (vascular endothelial growth factor-A) which is important for vascularization, were summarized for primary tumors and distant metastases [42]. Previous work suggested a predominant association of tumor dormancy with cancer stem cells and metastatic cancer cells, which promote late recurrences [43]. Moreover, cancer stem cells can disseminate to distal organs early in tumor development [44] and this suggests widespread and selective recurrence after dormancy. Thus, BMP signaling selectively relays metastatic relapse of breast cancer cells to the lung [45]. Further dormancy-associated genes include the Wnt/β-catenin signaling cascade [46]. In addition, TGF-β plays an important role during metastasis and dormancy [47,48]. All of these genes were markedly altered in MDA-hyb5 versus MDA-MB-231. In contrast, only some differences in these gene expressions were detectable in MDA-hyb1 versus MDA-MB-231 cells or in MDA-hyb3 versus MDA-MB-231 cells (Figure 5B). Quantitative PCR analysis was performed for these putatively dormancy-related genes and likewise demonstrated modified expression levels (Figure 5C). A principal component (PC) analysis of the examined genes revealed alterations among the four cell lines. In particular, significant differences were displayed by MDA-hyb5 cells supporting potentially altered tumorigenic behavior (Figure 5D).

**Figure 6:**
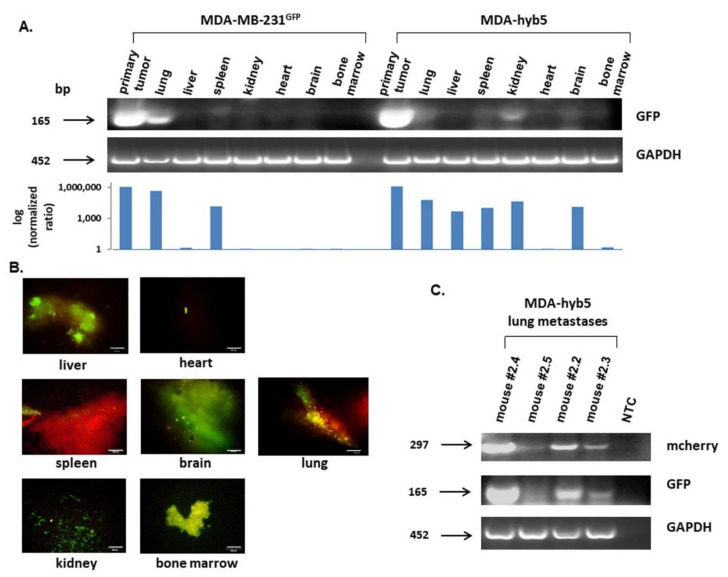
(**A**) Following the dissection of MDA-MB-231^GFP^ control cells- and MDA-hyb5 (mouse #2.5) cells-induced primary tumors and distal organ metastases including lung, liver, spleen, kidney, heart, brain, and bone marrow (prepared from femoral content), RNA aliquots were subjected to RT-PCR for GFP. GAPDH transcripts served as an equal loading control. Normalization of GFP expression levels by the corresponding GAPDH ratios was performed by densitometry scanning using the image J software. The resulting quantitatively comparable GFP expression levels in the different organs are demonstrated as a logarithmic bar diagram in the lower panel. (**B**) Distal organs and bone marrow from mouse #2.2 were dissected after euthanasia and evaluated by fluorescence microscopy, whereby appropriate fluorescence should indicate the formation of distant metastases. Bars represent 100 µm. (**C**) Aliquots of lung metastases from all four MDA-hyb5-induced mouse tumors, including a corresponding no template control (NTC), were subjected to RT-PCR for mcherry and GFP detection. GAPDH transcripts served as an equal loading control.

While MDA-hyb5 cells rapidly developed tumors after an initial dormancy-like phase of up to about 6 months, we were interested in the metastatic behavior of these cancer hybrid cells. Cancer cell-expressing GFP, although weakly visible in some bands compared to the strong primary tumor, was detectable in all tissue aliquots of MDA-MB-231 control cells and MDA-hyb5 cells by RT-PCR (Figure 6A). The quantification of GFP levels after image J densitometry and the normalization by the corresponding GAPDH expression revealed a markedly higher metastatic potential of MDA-hyb5 cells when compared to the parental MDA-MB-231 cell line (Figure 6A, bar diagram). Moreover, thin sections of organ tissues and bone marrow were evaluated by fluorescence microscopy and likewise revealed GFP-carrying cancer cells in all investigated organs (Figure 6B). Since different MDA-hyb5-induced mice were examined by RT-PCR (mouse #2.5) and by fluorescence microscopy (mouse #2.2), these data suggested the presence of organ metastases in all tumor-carrying MDA-hyb5 mice. Indeed, lung tissues from the four MDA-hyb5 tumor-bearing mice were analyzed by RT-PCR for mcherry and GFP, respectively, and both transcripts were detectable in the lung tissues (Figure 6C).

In summary, these findings suggested a more rapid tumor development after escape from the initial dormancy in MDA-hyb5 cells when compared to the parental MDA-MB-231. Although molecular triggers to overcome this initial quiescence require further investigation, the expression levels of distinct genes associated with tumor dormancy were elevated in MDA-hyb5 cells. Moreover, the initiated speedy tumor growth of triggered MDA-hyb5 cells was paralleled by a simultaneously rapid development of organ metastases in all tumor-bearing mice.

**Figure 7:**
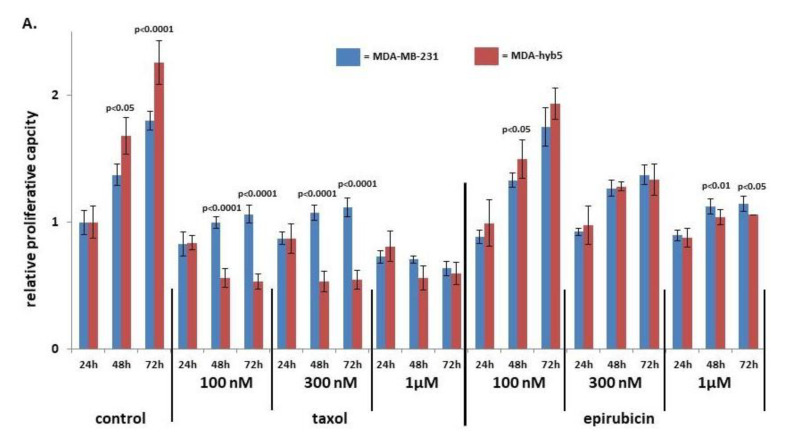

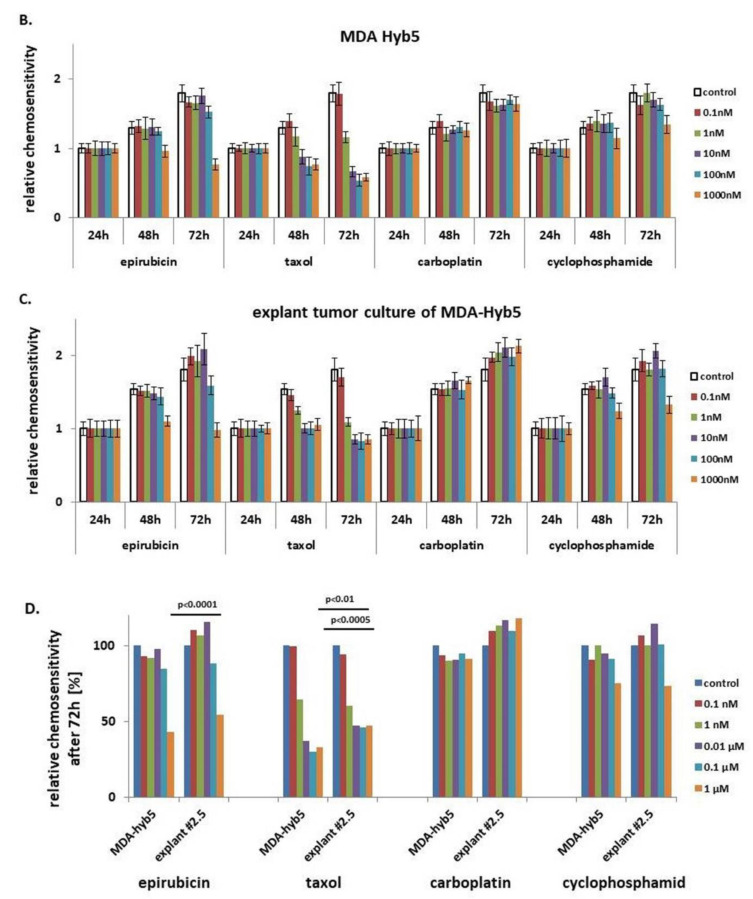
(**A**) MDA-MB-231 and MDA-hyb5 cells were treated with the indicated concentrations of taxol and epirubicin for 24 h to 72 h, respectively. Thereafter, the cells were lyzed and measured by fluoroscan assay. The data were normalized to the 24 h control cells. The relative data represent the mean ± s.d. of three independent experiments with three replicates each. Significance (*p*) was calculated by unpaired student’s *t*-test. (**B**) MDA-hyb5 cells and (**C**) explant cells from cultured MDA-hyb5-induced tumor #2.5 after dissection were exposed to different chemotherapeutic agents for up to 72 h. Untreated control cells (white bars on the left) were followed by the constant increase in drug concentrations between 0.1 nM and 1 µM (colored bars). After treatment with the corresponding drug at the indicated concentration and incubation time, the cells were lyzed and measured by fluoroscan assay. Data were normalized and relative fluorescence correlated to relative proliferative capacity as previously described [49]. Data represent the mean ± s.d. of three independent experiments with three replicates each. (**D**) Relative 72 h chemosensitivities for the different drug cytotoxicities were compared between MDA-hyb5 cells and explant cells from cultured mouse tumor #2.5 after dissection. Significance (*p*) was calculated by unpaired student’s *t*-test.

This aggressive tumor development raised further questions about potential chemotherapeutic approaches. Sensitivities to different concentrations of taxol and epirubicin were compared between parental MDA-MB-231 and MDA-hyb5 cells. While the proliferation of MDA-hyb5 control cells was significantly enhanced, low concentrations of 100 nM and 300 nM taxol inhibited MDA-hyb5 cell growth more than MDA-MB-231 cells (Figure 7A). However, taxol cytotoxicity displayed a plateau in MDA-hyb5 cells which suggests a constant level of resistance. Higher concentrations of 1 µM epirubicin also demonstrated more sensitivity to MDA-hyb5 cells (Figure 7A).

Further chemotherapeutic analysis was performed with explant cultures from dissected MDA-hyb5 tumor tissue. The outgrowing explanted cancer cells in passage two and in vitro growing MDA-hyb5 cells were subjected to different chemotherapeutic treatments. Whereas low drug concentrations of 0.1 nM and 1 nM displayed little if any effects, incubation with epirubicin and taxol at 0.01 µM, 0.1 µM, and 1 µM for 48 h up to 72 h revealed a concentration-dependent cytotoxicity. Epirubicin treatment reached about 57% in reduction in MDA-hyb5 cells and about 45% in decline in explant tumor cells, respectively, whereby populations formed a plateau (Figure 7B,C). Moreover, taxol-mediated cytotoxicity revealed about 67% in MDA-hyb5 and about 53% in the corresponding tumor explant cultures. Additionally, exposure to 1 µM cyclophosphamide demonstrated about 25% cytotoxicity in MDA-hyb5 cells, which was similar in explant tumor cells by about 26% after 72 h (Figure 7B,C).

A more detailed comparison of chemotherapeutic effects with the various drugs revealed little if any difference for epirubicin and carboplatin between MDA-hyb5 cells and the tumor explant culture cells, respectively (Figure 7D). In contrast, a significantly increased sensitivity for taxol and cyclophosphamide became detectable in the tumor explant culture cells which was observed after 48 h and 72 h, respectively (Figure 7B–D).

Taken together, these findings suggested a partially enhanced sensitivity of MDA-hyb5 cells to taxol and epirubicin when compared to the parental MDA-MB-231 cells. However, the observed maintenance of a certain basal proliferative level in MDA-hyb5 cells regardless of the applied drug concentration indicated the presence of potentially resistant cancer sub-populations. Moreover, the acquisition of partially reduced in vitro sensitivity against epirubicin and taxol in the tumor explant cultures indicated ongoing changes in biological properties during in vivo tumor development.

## 3. Discussion

Cell fusion can be part of both physiological and pathophysiological mechanisms [50]. The orchestration of signaling to enable cell fusion strongly depends on the cellular microenvironment and may involve alternative kinase pathways, e.g., to relay signals for differentiation or apoptosis [51]. Thus, cell fusion processes can contribute to tissue regeneration, such as the liver [52]. While cell–cell merger can occur by engulfment of a target cell, e.g., via cannibalism [53] or entosis-like mechanisms [54] including emperipolesis, which includes degradation of the target cell genome, fusion-associated MSC hybrid cell formation may be accompanied by a recombination of genomic parts from both parental donors in a nuclear hetero-to-synkaryon transition during subsequent cell divisions. Since aberrant DNA profiles or aneuploidy can also arise during abnormal cell division such as endoreplication, endomitosis, therapy-induced polyploidization, horizontal/lateral gene transfer, or deregulated cytokinesis, cancer cell fusion is observed in a variety of different cancer types and derived cancer cell lines [55]. 

Although most tumor-generated fusion cells are unable to survive, the remaining hybrid cancer cells can develop a proliferation advantage by overgrowing other cancer cells and eventually representing the majority of a population within a tumor tissue. Moreover, hybrid cancer cells after fusion with MSC can acquire stem cell properties. These effects underscore the significance of tumor fusion mechanisms to alter DNA content and contribute to the severity and worsening of primary tumors and metastatic growth [56,57].

Cell fusion mediated aneuploidy causes DNA instabilities and generates aberrant hybrid cells, which may be repaired or eliminated by a post-hybrid selection process [33]. A PHSP is associated with chromosomal reduction or reorganization which is required to enable survival of a genetically (meta-)stabilized phenotype after cell-cell merger such as fusion. Therefore, hybrid cell functionalities are determined partially by a multistep program of a PHSP [33,58]. Accordingly, the acquired new properties of MDA-hyb5 cells suggest that, besides fusion, at least part of this phenotype may represent a consequence of a PHSP during clonal conversion and expansion of the hybrid cancer cells.

Alterations in the DNA profile after fusion causes different properties and tumorigenic behavior in the cancer hybrid cells. While lentiviral transduction processes of the parental MDA-MB-231 and MSC populations themselves already could cause some changes, which are unrelated to the fusion process, cancer hybrid cell alterations were identified by comparison to the fluorophore-labeled parental cell populations rather than to wild-type cells. Accordingly, fusion-associated changes were substantiated by the different MDA-MSC-hybrid cell populations after the spontaneous cell merger of MDA-MB-231^cherry^ with different MSC^GFP^. All breast cancer hybrid populations (MDA-hyb1 to MDA-hyb5) demonstrated significantly increased proliferative capacity when compared to the parental MDA-MB-231^cherry^ cells. Moreover, tumor development and formation of metastases also displayed marked differences. Previous work demonstrated that MDA-hyb1 and MDA-hyb2 cells developed a rapidly enhanced tumor growth and metastases compared to MDA-MB-231 cells [11]. Conversely, MDA-hyb3 and MDA-hyb4 cells also started tumor development after initial subcutaneous injection of the cancer hybrid cells. However, the growth of tumors progressed much more slowly when compared to MDA-MB-231 cells. Furthermore, cancer cell spreading to distal organs was limited during MDA-hyb3 tumor induction and revealed no detectable metastases in lung and kidney, respectively [32]. In contrast, MDA-hyb5 cells displayed a completely different tumor behavior. Neoplastic tissue by MDA-hyb5 cells became detectable when tumor growth and metastatic development of MDA-MB-231 cells were already terminated. However, when MDA-hyb5 cells initiated tumor growth, the progressive development of primary tumors and distal metastases was significantly accelerated when compared to MDA-MB-231 cells. This suggested a rapid tumor neovascularization and enhanced dissemination of MDA-hyb5 cells to enable development of organ metastases nearly twice as fast as MDA-MB-231 cells.

The retarded development of the four primary tumors and associated organ metastases induced by MDA-hyb5 cells between 49 days and 161 days post subcutaneous injection indicated dormancy-like states. Dormancy of cancer cells describes a reversible cell cycle arrest similar to a previously suggested G_0′_ arrest cycle in a differentiation/retrodifferentiation program [59,60], whereby cells initially enter a transient quiescence. Asymptomatic cell populations can carry dormant cancerous lesions [61] whereby the majority of these lesions never progress to the stage of exponential tumor growth as controlled by hormones and the immune response. Cancer cells may require a temporary G_0′_ arrest cycle of dormancy for adaptation to altered microenvironmental tissue conditions, for coping with chemotherapeutic exposure, or for the acquisition of new mutations [62]. In particular, disseminated cancer cells with the potential to metastasize to distant tissues and organs may enter a state of dormancy by moving into a previously suggested transient G_0′_ arrest cycle to allow for the adaptation of functionality and metabolism to the new environment.

Although the trigger to awaken MDA-hyb5 cells after at least 161 days for progression of tumor development remains unclear, different factors were identified for potential escape from dormancy. A reasonable model for cancer dormancy also reflects the bone marrow as a predominant location for metastases [63]. Previous work suggested that the transition of breast cancer cells to dormancy involves accumulation of thrombospondin-1, which can function as an inhibitor of angiogenesis. For the vice versa case, regained proliferative capacity of the different cancer cell populations is accompanied by neovasculature sprouting and perivascular release of various growth factors [64]. In contrast to the angiogenesis-associated and neovascularization-associated factor VEGF-A displaying significant changes among the hybrid populations, transcripts of the thrombospondin-1 gene were not markedly altered in MDA-hyb5 cells as compared to MDA-MB-231 cells. However, multiple coordinated signals may be required to maintain cancer cells in quiescence, with subsequent triggers to escape the transient G_0′_ arrest cycle and to regain proliferative capacity for tumor development/recurrence. Taken together, these findings underscore the marked differences of MDA-hyb5 cells as compared to the other hybrids including the initial dormancy-like state of MDA-hyb5 cells which appears unique among the several investigated hybrid breast cancer populations.

Changes in tumor behavior and altered metastatic capacity in cancer hybrid cells are also associated with differences in chemotherapeutic response. The observed unresponsiveness beyond certain concentrations of chemotherapeutic agents indicated a potential resistance within the MDA-hyb5 population. On the other hand, permanent proliferation with self-renewal capacity and enhanced resistance to apoptotic stimuli, including anti-cancer drugs, are also features of cancer progenitor cells, tumor-initiating cells, and cancer stem cells [65,66]. This is also supported by the elevated resistance acquired by MDA-hyb5 tumor explant cells against taxol and epirubicin and suggests further functional modifications of the cancer hybrid cells during the rapid tumor development and accounts for instability and an increased heterogeneity. These findings further suggested the prescence of unstable in vivo phenotypes of MDA-hyb5 cells with continuous alterations, which would complicate successful therapeutic regimens.

## 4. Materials and Methods

### 4.1. Cell Culture

Human MDA-MB-231 breast cancer cells were obtained from the ATCC (#HTB-26) and cultivated initially at 1500 cells/cm^2^ in Leibovitz’s L-15-medium (Invitrogen Life Technologies, Carlsbad, CA, USA) supplemented with 10% (*v*/*v*) fetal bovine serum, 100 U/mL penicillin, 100 µg/mL streptomycin, and 2 mM L-glutamine (Sigma Chemie GmbH, Taufkirchen, Germany). The subculture of MDA-MB-231 cells was performed by trypsin/EDTA (Biochrom GmbH, Berlin, Germany) treatment for 5 min at 37 °C.

Primary hUC-MSC030816 were isolated from umbilical cord tissue explant cultures as described previously for other MSC [67]. Briefly, hUC-MSC were cultured in MSC growth medium (αMEM (Sigma Chemie GmbH, Steinheim, Germany) supplemented with 10% allogeneic human AB-serum, 100 U/mL penicillin, 100 µg/mL streptomycin, and 2 mM L-glutamine (Sigma Chemie GmbH, Taufkirchen, Germany)) and subculture in passages (*p*) was performed following treatment with Accutase (Capricorn Scientific GmbH, Ebsdorfergrund, Germany) at 37 °C for 3 min. Usage of hUC-MSC was approved by the Ethics Committee of Hannover Medical School, project #443 on 26 February 2009 and informed written consent was obtained from each patient.

All MDA hybrid cell lines (MDA-hyb1 to MDA-hyb5) were cultured in xeno-free conditions in MSC growth medium (αMEM (Sigma Chemie GmbH) supplemented with 10% allogeneic human AB-serum (blood from male AB donors was commercially obtained from the blood bank at Hannover Medical School, Germany, and processed to serum), 100 U/mL penicillin, 100 µg/mL streptomycin, and 2 mM L-glutamine (Sigma Chemie GmbH). Subculture in passages (*p*) was performed following mechanical detachment of the loosely adherent MDA-hyb1 cells and by TrypLE (Life Technologies GmbH, Darmstadt, Germany) treatment of MDA-hyb2, MDA-hyb3, MDA-hyb4, and MDA-hyb5 cells at 37 °C for 3 min, respectively.

All cell lines were tested for mycoplasma by the luminometric MycoAlert Plus mycoplasma detection kit (Lonza Inc., Rockland, ME, USA) according to the manufacturer’s recommendations. Authentication of the different cell lines was performed by short tandem repeat (STR) fragment analysis using the GenomeLab human STR primer set (Beckman Coulter Inc., Fullerton, CA, USA) [39]. The identity of STR fragments was confirmed for MDA-MB-231 cells according to the STR database provided by the ATCC, Manassas, VA, USA and for MDA-hyb1 and MDA-hyb2 cell lines as outlined in previous work [11]. For stable fluorescence labeling, hUC-MSC and MDA-MB-231 cells were transduced with a third generation lentiviral SIN vector containing the eGFP or the mcherry gene, respectively, as reported elsewhere [37].

### 4.2. In Vitro Proliferation and Cytotoxicity Measurements

The proliferation rate was determined by fluorescence measurement using the fluoroscan assay as previously described [12]. Briefly, 1000 cells/well of MDA-MB-231^cherry^, MDA-hyb1, MDA-hyb3, or MDA-hyb5 populations were plated in flat bottom 96-well plates (Nunc/ThermoFischer Scientific, Roskilde, Denmark) with (200 μL/well) standard culture medium.

For cytotoxicity measurements with different chemotherapeutic agents, 1000 cells/well of MDA-MB-231 cells, MDA-hyb5 cells, or explant culture cells from MDA-hyb5-derived mouse tumors were plated in flat bottom 96-well plates (Nunc/ThermoFischer Scientific) with (100 μL/well) standard culture medium. The explant culture cells from MDA-hyb5-derived mouse tumors were obtained by an isolation procedure as extensively described elsewhere for primary human breast cancer epithelial cells [68]. Isolated explant culture cells were subcultured with TrypLE (Life Technologies GmbH) and used in P2 for cytotoxicity measurements. Following overnight attachment of the different cultures, 100 µL of drug solvent in the culture medium was added as a control and in further wells 100 µL of culture media containing appropriate dilutions of epirubicin, taxol, carboplatin, or cyclophosphamide were respectively added to the cells.

After incubation for up to 72 h at the appropriate time points, the medium was removed and the cells were lysed with 5% (*w*/*v*) SDS. Thereafter, the fluorescence intensities of GFP or cherry cell homogenates, which corresponded to the appropriate cell number of cancer cells, were measured at an excitation of 485 nm and an emission of 520 nm (GFP) or an excitation of 584 nm and an emission of 612 nm (cherry) using the Fluoroscan Ascent Fl (ThermoFisher Scientific, Schwerte, Germany).

### 4.3. In Vivo Experiments

Animal research using NODscid mice was performed by following the internationally recognized guidelines on animal welfare. The project has been approved by the institutional licensing committee (Niedersächsisches Landesamt für Verbraucherschutz und Lebensmittelsicherheit, Oldenburg, Germany) ref. # 33.19-42502-04-15/1992 on 17 December 2015.

About 2 × 10^6^ human MDA-MB-231^GFP^ and MDA-hyb5 breast cancer cells were subcutaneously injected into the left shoulder of five animals from 5 to 6 weeks old female NODscid mice, respectively. The mice were examined and weighted two times per week. Tumors smaller than 1 mm^3^ induced by MDA-MB-231^GFP^ cells became detectable between 10 and 18 days. After 32 days following MDA-MB-231^GFP^ cell transplantation, the five tumor-bearing animals were sacrificed by cervical dislocation following the criteria for termination of the experiment. Tumors induced by MDA-hyb5 cells were determined in four mice subsequently at later and different time points between 49 days and 161 days. One mouse remained without detectable tumors after 180 days of MDA-hyb5 cell injection.

Primary tumor tissues were isolated, washed in PBS, and weighted. In culmination with the dissected organs, which included the lung, liver, spleen, kidney, heart, and brain, the tissues were washed in PBS, examined by fluorescence microscopy for the presence and accumulation of metastatic cells, shock-frozen in liquid nitrogen, and stored at −80 °C for further analysis. Bone marrow was harvested by cutting the femur and rinsing the open bone with PBS followed by centrifugation (360 g/7 min) of the bone marrow cells.

### 4.4. Transcript Analysis by PCR

Total RNA was isolated from the tumor tissues and the organs using RNeasy Mini Kit (Qiagen, Hilden, Germany) according to the manufacturer’s instructions. One µg of RNA was reverse-transcribed into cDNA and reactions were performed with corresponding RT-PCR primers:

⁃ mcherry (sense: 5′-TTC ATG TAC GGC TCC AAG GC-3′; antisense: 5′-CTG CTT GAT CTC GCC CTT CA-3′; amplification product 297 bp);
⁃ eGFP (sense: 5′-CTA TAT CAT GGC CGA CAA GCA GA-3′; antisense: 5′-GGA CTG GGT GCT CAG GTA GTG G-3′; amplification product 165 bp);
⁃ CD73 (sense: 5′-CGC AAC AAT GGC ACA ATT AC-3′; antisense: 5′-CTC GAC ACT TGG TGC AAA GA-3′; amplification product 241 bp) [37];
⁃ CD90 (sense: 5′-GGA CTG AGA TCC CAG AAC CA-3′; antisense: 5′-ACG AAG GCT CTG GTC CAC TA-3′; amplification product 124 bp);
⁃ CD105 (sense: 5′-TGT CTC ACT TCA TGC CTC CAG CT-3′; antisense: 5′-AGG CTG TCC ATG TTG AGG CAG T-3′; amplification product 378 bp);
⁃ GAPDH as a control (sense: 5′-ACC ACA GTC CAT GCC ATC AC-3′; antisense: 5′-TCC ACC ACC CTG TTG CTG TA-3′; amplification product 452 bp) [69] (all primers are customized by Eurofins, MWG GmbH, Ebersberg, Germany).

Aliquots of 25 µL of each RT-PCR product were separated on a 2% agarose gel, including the standard GeneRuler 100 bp DNA Ladder (Thermo Scientific) and visualized by GelRedTM (Biotium Inc., Hayward, CA, USA), staining specifically as described previously [70].

For qPCR, the following primers were used:

⁃ Tumor necrosis factor receptor superfamily member 8 (TNFR SF8, CD30) (sense: 5′-ATC TGT GCC ACA TCA GCC ACC A-3′; antisense: 5′-AAG GTG GTG TCC TTC TCA GCC A-3′; amplification product 110 bp);
⁃ Tumor necrosis factor receptor superfamily member 1B (TNFR SF1B) (sense: 5′-CGT TCT CCA ACA CGA CTT CAT CC-3′; antisense: 5′-ACG TGC AGA CTG CAT CCA TGC T-3′; amplification product 102 bp);
⁃ Bone morphogenic protein 1 (BMP1) (sense: 5′-GCC TGT GCT GGT ATG ACT ACG-3′; antisense: 5′-CAT CTG GGT AAT TGG GCG ATT GG-3′; amplification product 243 bp);
⁃ Bone morphogenic protein 7 (BMP7) (sense: 5′-ACC AGA GGC AGG CCT GTA AGA-3′; antisense: 5′-CTC ACA GTT AGT AGG CGG CGT AG-3′; amplification product 108 bp);
⁃ Transforming growth factor-beta3 (TGF-b3) (sense: 5′-CTA AGC GGA ATG AGC AGA GGA TC-3′; antisense: 5′-TCT CAA CAG CCA CTC ACG CAC A-3′; amplification product 161 bp);
⁃ Vascular cell adhesion molecule-1 (VCAM-1) (sense: 5′-GAT TCT GTG CCC ACA GTA AGG C-3′; antisense: 5′-TGG TCA CAG AGC CAC CTT CTT G-3′; amplification product 118 bp);
⁃ Glyceraldehyde 3-phosphate dehydrogenase (GAPDH) (sense: 5′-GTC TCC TCT GAC TTC AAC AGC G-3′; antisense: 5′-ACC ACC CTG TTG CTG TAG CCA A-3′; amplification product 131 bp);
⁃ Ribosomal Protein L13a (RPL13A) (sense: 5′-CTC AAG GTG TTT GAC GGC-3′; antisense: 5′-TAC TTC CAG CCA ACC TCG-3′; amplification product 143 bp).

The QuantiTect SYBR green PCR kit (Qiagen) was used according to the manufacturer’s instructions and PCR was performed in quadruplicates using a CFX384 touch real time detection system (Bio-Rad Laboratories GmbH, Feldkirchen, Germany) and analyzed using the BioRad CFX manager software (V.3.1.1517.0823) (Bio-Rad Laboratories, Inc., Hercules, CA, USA). Normalization of the genes analyzed by qPCR GAPDH and RPL13A were used as housekeeping genes. Melting curves were performed to verify primer quality. High deviation replicates were excluded. The log2 normalized expression values were used for statistical analysis and for generating principal components using GraphPad Prism v9.00 (GraphPad Software, San Diego, CA, USA).

### 4.5. Microarray-Based mRNA Expression Analysis (Single Color Mode)

The mRNA microarray analyses were performed as extensively described elsewhere [11] with minor modifications. The Microarray utilized in this study represents a refined version of the Whole Human Genome Oligo Microarray 4 × 44 K v2 (Design ID 026652, Agilent Technologies Deutschland GmbH, Waldbronn, Germany), named “026652QM_RCUG_HomoSapiens” (Design ID 084555), developed by the Research Core Unit Genomics (RCUG) of Hannover Medical School. Microarray design was created at Agilent’s eArray portal using a 1 × 1 M design format for mRNA expression as a template. All non-control probes of design ID 026,652 have been printed five times within a region comprising a total of 181,560 Features (170 columns × 1068 rows). Four of such regions were placed within one 1 M region giving rise to four microarray fields per slide to be hybridized individually (Customer Specified Feature Layout). Control probes required for proper Feature Extraction software operation were determined and placed automatically by eArray using the recommended default settings.

Synthesis of Cy3-labeled cRNA was performed with the “Quick Amp Labeling kit, one color” (#5190-0442, Agilent Technologies Deutschland GmbH) using 500 ng of total RNA as input and in accordance to the manufacturer’s recommendations, with the exception that reaction volumes were halved. cRNA fragmentation, hybridization, and washing steps were carried out as recommended in the “One-Color Microarray-Based Gene Expression Analysis Protocol V5.7”, with the exception that 3000 ng of labelled cRNA were used for hybridization. Slides were scanned on the Agilent Micro Array Scanner G2565CA (pixel resolution 3 µm, bit depth 20). Data extraction was performed with the “Feature Extraction Software V10.7.3.1” using the extraction protocol file “GE1_107_Sep09.xml”.

The MDA-MB-231^cherry^ cells were investigated together with the cancer hybrid populations MDA-MSC-hyb1, MDA-MSC-hyb3, and MDA-MSC-hyb5. Alterations in transcript levels were compared displaying a more than 2-fold difference in gene expression. Microarray data are stored at the NCBI-GEO database with the accession no. #GSE157199 (filed on 28 August 2020).

## 5. Conclusions

Characteristics of the breast cancer hybrid cell line MDA-hyb5 demonstrated initial quiescence in vivo. Following release from dormancy, the cells developed tumors and metastases much faster than the parental breast cancer cells. Although the trigger for tumor growth in MDA-hyb5 cells requires further elucidation, the signals for initiated proliferation of these cancer hybrid populations enlarge tumor plasticity and counteract successful interventional strategies. Consequently, the generation and expansion of different cancer hybrid populations in vivo would be associated with bad patient prognoses by frequently ongoing changes and acquisition of new cancer cell functions.

While permanent alterations in cancer progenitor cells and cancer stem cells promote therapy resistance and disease progression [71], the appearance of cancer hybrid cells, e.g., by MSC/breast cancer cell fusion contributes to elevated cancer cell plasticity. Moreover, the generation of new cancer hybrid cell populations by cell fusion diversifies tumorigenic properties and chemotherapeutic responsiveness, which markedly increases tumor heterogeneity. On the other hand, detailed molecular studies using models of initial tumor quiescence enable further insights into tumor dormancy and subsequent tumor initiation also providing a better understanding of mechanisms which trigger tumor relapse and recurrence of metastases.

## Figures and Tables

**Table 1:** Analysis of tumor development in MDA-MB-231 and MDA-hyb5 tumors.

Cells (#Mouse)	Tumor Detection (Day)	Tumor Dissection (Day)	Difference Between Tumor Detection and Dissection (Days)
MDA-MB-231 (#1.2)	10	32	22
MDA-MB-231 (#1.5)	10	32	22
MDA-MB-231 (#1.1)	14	32	18
MDA-MB-231 (#1.4)	14	32	18
MDA-MB-231 (#1.3)	18	32	14
			
MDA-hyb5 (#2.4)	49	59	10
MDA-hyb5 (#2.5)	70	81	11
MDA-hyb5 (#2.2)	122	131	9
MDA-hyb5 (#2.3)	161	173	12

# refers to the nomenclature of the corresponding mouse.

## Data Availability

Not applicable.

## Abbreviations

BMP	bone morphogenic protein
CXCR4	C-X-C motif chemokine receptor 4
FACS	fluorescence-activated cell sorting
GFP	green fluorescent protein
hUC	human umbilical cord
MDA-hyb5	MDA-MB-231^cherry^/MSC030816^GFP^-hybrid cells (=MDA-MSC-hyb5)
MSC	mesenchymal stroma/stem-like cells
PHSP	post-hybrid selection process
TGF-β	transforming growth factor-beta
TNF	tumor necrosis factor
VCAM-1	vascular cell adhesion molecule-1
VEGF-A	vascular endothelial growth factor-A
